# An Algorithm to Automate Yeast Segmentation and Tracking

**DOI:** 10.1371/journal.pone.0057970

**Published:** 2013-03-08

**Authors:** Andreas Doncic, Umut Eser, Oguzhan Atay, Jan M. Skotheim

**Affiliations:** 1 Department of Biology, Stanford University, Stanford, California, United States of America; 2 Department of Applied Physics, Stanford University, Stanford, California, United States of America; Texas A&M University, United States of America

## Abstract

Our understanding of dynamic cellular processes has been greatly enhanced by rapid advances in quantitative fluorescence microscopy. Imaging single cells has emphasized the prevalence of phenomena that can be difficult to infer from population measurements, such as all-or-none cellular decisions, cell-to-cell variability, and oscillations. Examination of these phenomena requires segmenting and tracking individual cells over long periods of time. However, accurate segmentation and tracking of cells is difficult and is often the rate-limiting step in an experimental pipeline. Here, we present an algorithm that accomplishes fully automated segmentation and tracking of budding yeast cells within growing colonies. The algorithm incorporates prior information of yeast-specific traits, such as immobility and growth rate, to segment an image using a set of threshold values rather than one specific optimized threshold. Results from the entire set of thresholds are then used to perform a robust final segmentation.

## Introduction

The analysis of behavior in individual cells is essential to understand cellular processes subject to large cell-to-cell variations. Bulk measurements and cell synchronization methods are insufficient to study such processes because a lack of synchrony masks oscillations, all-or-none effects, sharp transitions, and other dynamic processes operating within individual cells [1], [2], [3], [4], [5], [6], [7], [8]. The vast majority of all single cell studies ultimately relies on the ability to accurately segment and track cells. We here refer to *segmentation* as the process of separating regions of interest (cells) from background (non-cells) in an image [9]. Moreover, high quality data for studying dynamic processes can only be obtained if segmentation is coupled with the ability to *track* cells, *i.e.*, to correctly identify the same cell over consecutive time points in an experiment.

Segmentation of individual cells relies on the ability to detect cell boundaries and classify all pixels in a given image as ‘cell’ or ‘non-cell’ pixels. This differentiation is accomplished by specifying a threshold or a threshold function. There are several ways in which this threshold can be determined, ranging from simpler intensity based thresholds to usage of more complex functions such as graphical models [10], [11], pattern recognition [12], deformable templates [13], cell contours [14] or the watershed algorithm [15]. Despite these efforts, we still lack a unified approach that robustly detects all cells for all time points.

This is a major challenge in image analysis as the intensity of areas to be designated as ‘cell’ can vary significantly through time and even within the same cell due to varying cellular morphologies, imaging artifacts, and organelles. Moreover, most cells do not have an easily defined and constant geometrical form; therefore, fitting predefined objects on cells does not work in most cases. Besides, segmentation has to be very accurate for successful analyses of time-lapse microscopy, since the overall time-series segmentation success rate decreases geometrically with the length of the time series. For example, the probability of successfully tracking a cell for 100 time-points given a 99% segmentation success rate is only ∼37% ( = 0.99^100^ from assuming an identical and independently distributed segmentation probability).

To address these image processing problems for the widely-used model organism *Saccharomyces cerevisiae* (budding yeast), we developed a novel segmentation and tracking algorithm. Budding yeast is ideal for single cell time-lapse imaging studies because it combines considerable variation in key cell characteristics (protein levels and expression, cell size, shape, and age), with a short generation time and immobility [16], [17], [18]. So far, considerable progress has been made towards solving the yeast segmentation problem by refining algorithms for segmentation [10], [11], [13], [15], [19], [20], [21], [22], [23], as well as for tracking [11], [20], [22], [23]. Additional algorithms exist to characterize morphology [24], [25] and protein localization [12], [19], [20], [26]. However, we still lack a robust approach for the segmentation and tracking of budding yeast that is easy to implement and computationally efficient.

More specifically, our algorithm is based on the idea that summing multiple repeated segmentations of the same phase contrast image using sequentially varying thresholds is more robust than any algorithm based on a sole potentially optimized threshold. Such a strategy generates an unsupervised, and accurate final segmentation. We show that this method segments and tracks cells with different morphologies as well as cells within dense colonies with very high accuracy. We also present an example of how this algorithm can be used to determine specific cell cycle phases and dynamics. Our algorithm is fully automated following an initial manual seeding of the cells to be tracked. Moreover, the algorithm is easy to implement, and we have constructed a graphical user interface (GUI) to facilitate its application (see Supporting Information S1).

## Results

### Algorithm

**Figure 1:**
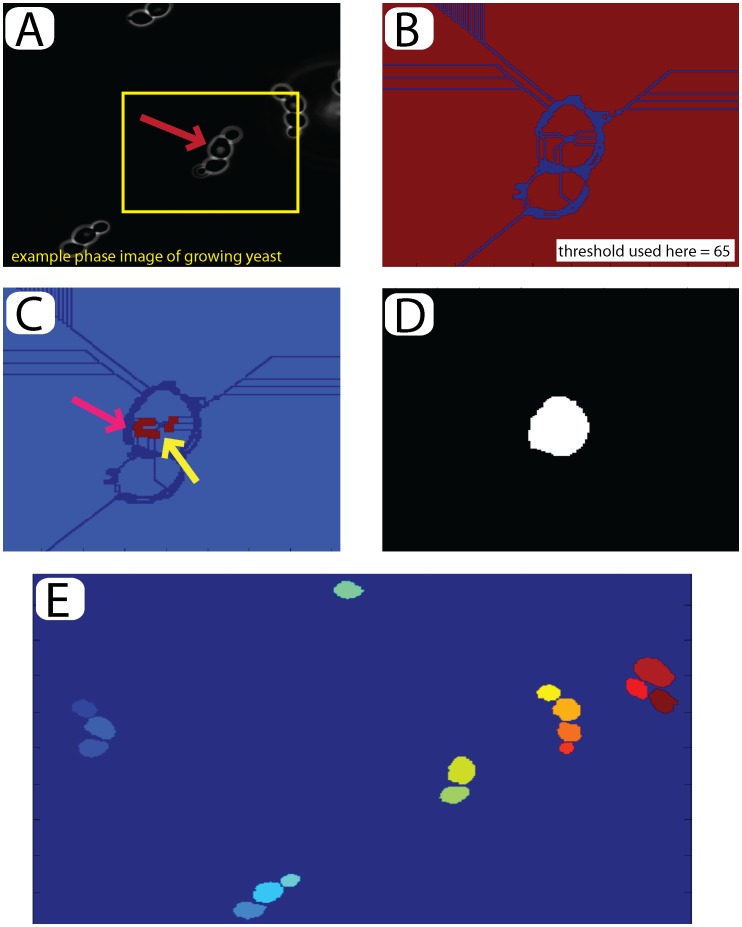
Seeding and initial selection: (A) Open the last image of the time-series to segment and track. An example cell is selected to build the initial seed (red arrow). (**B**) A watershed algorithm is applied to the image using a manually set threshold between 0 and 255 (threshold used here = 65). (**C**) Defects in the watershed are corrected manually. Subsections of the cell may be joined as indicated by the yellow arrow, wheras non-cell material may be removed as indicated by the pink arrow. Compare with the boxed region in (B). (**D**) The program automatically fills holes and one-pixel ‘cracks’ to complete the seed. (**E**) Final image for the entire field of view with 16 seeded cells inducated by their color.

**Figure 2:**
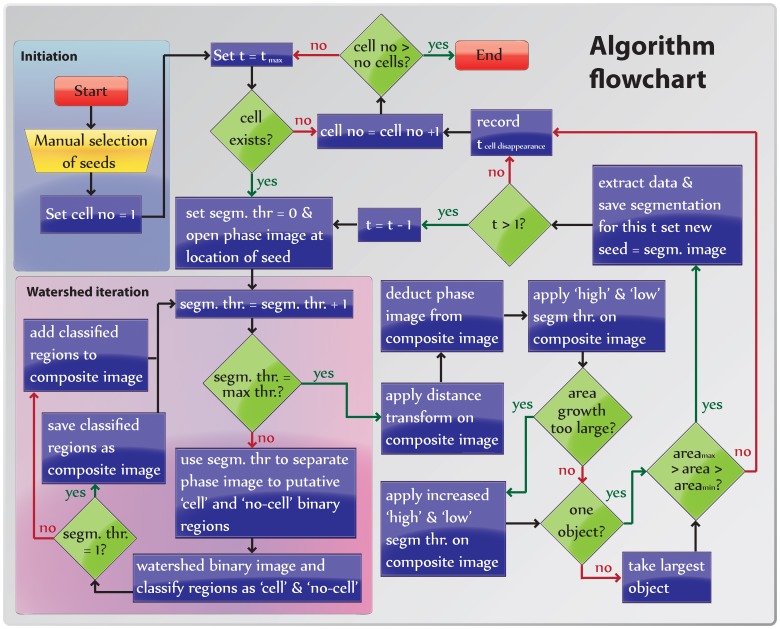
Flowchart of the image analysis algorithm (see text).

**Figure 3:**
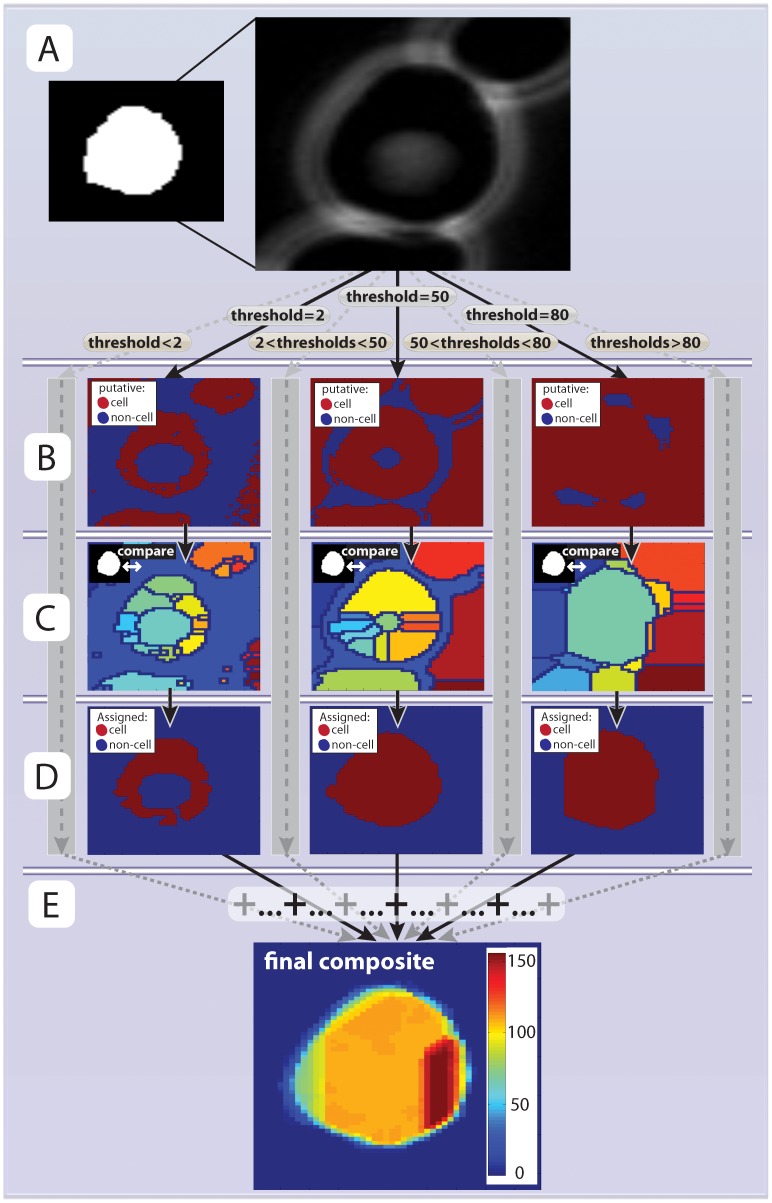
Segmentation. (**A**) A phase image region around the previously selected seed is opened. (**B**) The phase image is transformed into a series of binary images by applying thresholds such that any pixel that is lower than the threshold is assigned as ‘putative cell’ (red regions) or ‘non-cell’ (blue regions). In total 256 ( = 2^8^) thresholds are applied corresponsing to all possible values the phase image can take (example thresholds 2, 50 and 130 are shown here). (**C**) The watershed algorithm is applied to all thresholded images from (B). The result is a set of distinct regions. Each region is compared with the seed (inset) and the phase image and classified as ‘cell’ or ‘non-cell’. (**D**). All ‘cell’ areas, from all thresholds, are summed together (**E**). The composite image allows for more accurate segmentation than an optimized single threshold.

**Figure 4:**
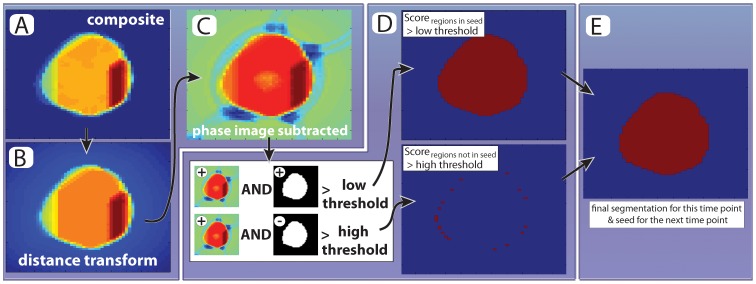
Final image thresholding. (**A,B**) First, we use the seed image to calcuate the euclidean distance from each non-cell pixel to the nearest cell-pixel and subtract it from the composite image (cf Figure 3e). This penalizes cell movements between frames. (**C**) The phase image multiplied by a constant is subtracted from the distance transformed image shown in B. (**D**) The intensity of each pixel from (C) is normalized and compared to a pair of thresholds. There are two conditions under which a pixel is assigned as part of the cell: (i) If the value is higher than the *low threshold* AND present in the seed or (ii) If the value is higher than the *high threshold* regardless of wheather the pixel is present in the seed. (**E**) All pixels fulfilling the two conditions are included in the final segmentation. Note that by allowing for ‘new’ pixels to be included in the cell, albeit at a higher threshold, we fulfill the need to balance changes in cell volume and slight cell movements with robust segmentation.

We here present the main outline of the algorithm. For a detailed step-by-step description of the algorithm see materials and methods section ‘algorithm outline’ and figures 1, 2, 3, and 4.

Before segmentation, images are typically processed in one or more steps such as filtering and rescaling, and then processed by a threshold function that differentiates ‘cell’ regions from ‘non-cell’ regions. The procedure of generating such a threshold function has proven a major challenge, as a specific threshold that might work for a given cell at some points in time and space might not necessarily work at other times and/or for other cells. In fact, it is not even certain that we can find a good threshold for any given cell since the intensity of boundaries and intercellular regions might vary significantly. Moreover, depending on the complexity of the threshold and pre-processing methods, the segmentation may take excessive processor time, be specific for each imaging pipeline, and require manual input.

To overcome these difficulties, we developed an algorithm that uses all possible thresholds to segment an image. Next, the algorithm uses a ‘plurality vote’ or sum of all segmentations to achieve a robust highly accurate final segmentation.

The algorithm is divided into three parts: First, cells are selected (seeded) semi-manually from the last time frame. Next, the seeds are segmented and tracked backwards in time, and, finally, the data obtained from the experiment are extracted and analyzed.

Segmenting backwards in time provides the following advantages: (i) As all cells are selected in the last frame, no subroutines are needed to identify newborn cells (buds). (ii) As all cells are present at the last time point, backward segmentation allows for the selection of only the cells of interest, avoiding uninteresting (*e.g.,* dead or newborn) cells. (iii) Since we begin segmenting cells at their presumed maximal size, we can set a bound on the upper cell size through the movie to prevent ‘out of control growth’ missegregations. We note that our algorithm relies on budding yeast not moving significantly between frames and requires that the cell growth rate is significantly smaller than the sampling frequency.

### Cell Seeding

Manual selection of all cells of interest in the last time point of interest (seeding) is required to initiate the algorithm. Any frame can be chosen as the last time point of interest. Note that we will use the ‘*first time point*’ to denote the first point we segment even though this is often the last time point in the experiment (t = t_max_). Similarly, the term ‘*last time point*’ refers to the last time point we segment, which most often is the first time point in the experiment (t = 0).

To select cells of interest, we segment the *first time point* semi-manually using a watershed algorithm with an adjustable threshold. Manual correction ensures accurate seeding and is facilitated by a graphical user interface (Figure 1a,b). The outcome of the seeding step is a seed image with all selected cells numbered from left to right (Figure 1c,d,e).

### Cell Segmentation and Tracking

Once the seed has been selected, cells are segmented one by one and tracked backwards through time. To segment images, we apply a watershed algorithm to all possible thresholds in the image (one for each possible level of brightness, here ranging from 0 to 255 as we are using 8bit images), and sum up the results (Figure 3a–d). The resulting composite image (Figure 3e) where each pixel has a value between 0 (segmented for no threshold) to 255 (segmented for all thresholds) (cf Figure3b and 4c, see also steps 1–10 in the algorithm description in the materials and methods sections). This approach resembles statistical bagging, where data is resampled and weighted to achieve a more robust model for estimation [27]. The difference is that the composite image/dataset is here generated systematically using all possible thresholds instead of by bootstrapping.

After the generation of the final composite image it is processed in two steps before segmentation. First, we calculate the Euclidean distance of a pixel to the nearest pixel in the previously segmented image and subtract it from the composite score image to penalize cellular movements (figure 4a,b). Next, the original phase image is subtracted from the composite score image (figure 4c). This prevents boundary regions (cf bright white in Figure 3a) from being scored as ‘cell’ (see also steps 11–13 in algorithm description in the materials and methods sections).

Next, the final composite image is segmented using two thresholds: one permissive for regions classified as ‘cell’ in the previous time-point, and one restrictive for regions that were not classified as ‘cell’ (steps 14–20 in algorithm description; Figure 4d,e). This approach has two main benefits: First, it balances the need to segment the entire cell with the natural shrinkage of the cell (as the segmentation runs backwards in time growing cells tend to get smaller); Second, it automatically tracks the cell. Note that these two thresholds are typically the only ones changed from experiment to experiment and they are easily adjusted upon inspection and do not require complicated optimization procedures. Once a cell has been segmented, the segmentation is stored and used as a seed for the next time point (steps 21–22 in the algorithm).

### Algorithm Performance

**Figure 5:**
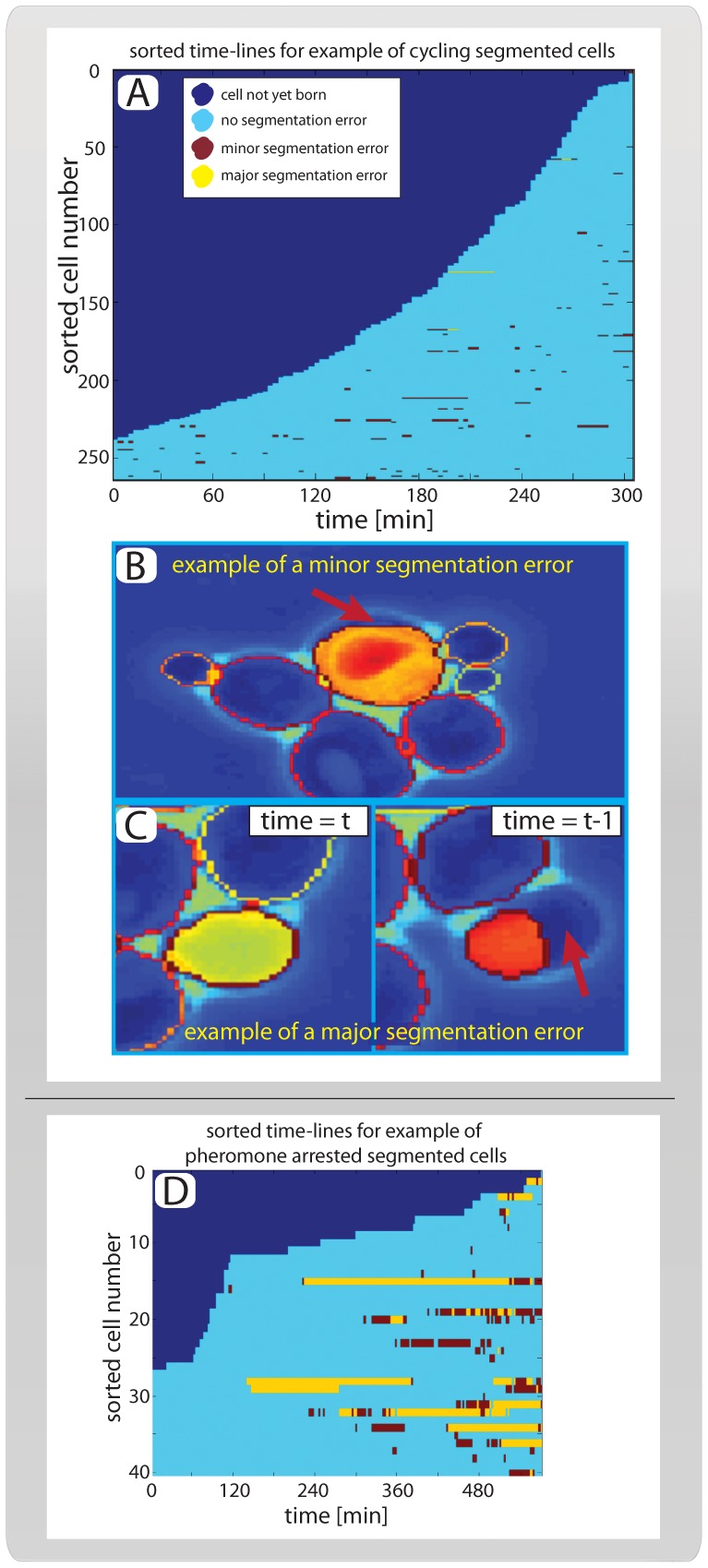
Performance. (**A**) Sorted cell traces for 263 example cells. All cells are present at the first (t_max_ = 300 min) segmented time point because the tracking and segmentation runs backwards in time. (**B**) Example of a minor segmentation error is indicated by the red arrow. Note that these errors are identified manually and represent cells whose area is segmented between 90% and 95% correctly. (**C**) Example of a major segmentation error, corresponding to a cell whose area is segmented less than 90% correctly as shown in the right panel. Note that the vast majority of major segmentation errors stem from unexpected cell movement. As seen here, the erroneously segmented cell moves significantly between the frames shown in the left and right panels. (**D**) Sorted cell traces for cells arrested in mating pheromone, colors indicate segmentation errors as in ‘A’.

To test the performance of our algorithm, we used a commercially available microfluidics system to grow budding yeast for 300 minutes in synthetic complete media containing 2% glucose [28]. Images were taken every three minutes. Next, phase images were exported and subsequently segmented and tracked using our algorithm. To avoid selection bias, we selected all available cells present in the last frame of the movie (first time point for the algorithm; t = 300 min). In total, 263 cells were selected in three fields of view, and 11727 segmentations were performed. On average, cells were present in the movie for 134 minutes or ∼45 frames with a standard deviation of 93 minutes (many cells are born near the end of the movie; Figure 5a). In total, segmentation took approximately 300 minutes corresponding to approximately 40 segmentations per minute using a computer with an Intel(R) core(TM) Quad CPU at 2.83 GHz and 4.00GB RAM running on a 32-bit windows vista operating system. As experiment itself took 300 minutes to perform, our algorithm can segment data in real time on a standard desktop computer.

To determine the quality of the segmentation, all cells were inspected manually and scored as being either properly segmented, having a minor error or having a major error (Figure 5). We defined a cell as ‘properly segmented’ if over 95% of its area was segmented correctly. We scored cells as having a ‘minor error’ if 90–95% of the area was correctly segmented and as having a ‘major error’ if less than 90% of the cell area was correctly segmented. The vast majority of all segmentation errors were classed as minor. Notably, we observed that major errors only occurred as a result of unexpected cell movements (Figure 5b,c; Table1; Movies S1, S2, S3). In total, 83% of all cells were segmented without any errors throughout the movie, and our error rate (major or minor) was less than 1 in 140 (Table 1).

**Table 1:** Algorithm performance on cycling cells and cells exposed to mating pheromone (see Figure 5 and text).

Segmentation statistics	Cycling cells	Low α-factor
Total number of segmented cells	263	40
Mean segmented frames per cell	44	140
Total number of segmentation events	11727	5657
Fraction of accurate segmentations	98.6%	88.1%
Fraction of minor segmentation errors	1.3%	4.2%
Fraction of major segmentation errors	0.1%	7.7%
Fraction of individual time-series without any errors	83.3%	37.5%

To test the effectiveness of the segmentation algorithm when faced with different cell shapes, we took advantage of the fact that yeast cells exposed to low concentrations of mating pheromone (α-factor), exhibit a pseudohyphal-like morphology characterized by elongated, polarized growth [29]. After 90 minutes, growing cells were exposed to a brief pulse (30 minutes) of high (240 nM) α-factor concentration, followed by a long period (450 minutes) of low (3 nM) α-factor concentration. We then applied our algorithm to segment and track the cells using the same metric as above (Figure 5d; Movie S4). As expected, the more irregular shape of the pheromone-arrested cells lowered the performance of the algorithm (see Table1). However, the performance remained acceptable as almost 90% of all segmentation events were classified as without error. In conclusion, this shows that our algorithm performs well on cells with several different morphologies.

### Extracting Features

**Figure 6:**
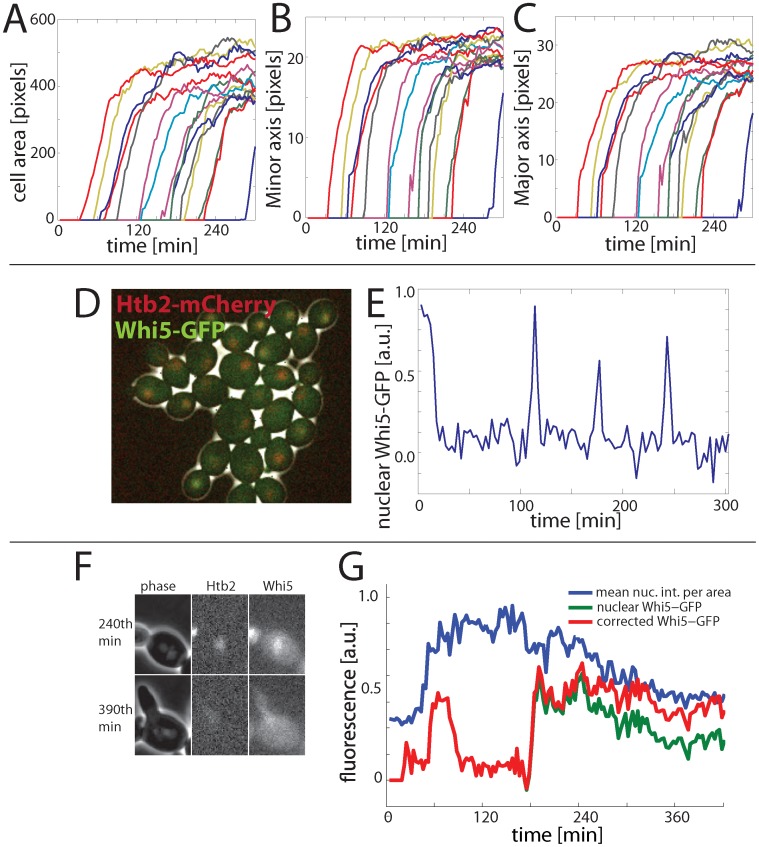
Example of fluorescence and morphological signal extraction. (**A–C**) Morphological parameters, here major/minor axis and cell area are calculated using our algorithm (the same colors represent the same cells in A–C). (**D**) Composite phase and fluorescence images of cells containing Whi5-GFP and Htb2-mCherry. (**E**) Example cell trace of nuclear Whi5-GFP. The nuclear Whi5-GFP is calcualted by fitting a 2D Gaussian to the brightest point for each cell after applying an averageing filter and assigning the brightest 25% as the nucleus. (**F**) Example of a cell whose nucleus is initially in focus at the 240^th^ minute, but subsequently goes out of focus. (**G**) Example trace of observed and corrected nuclear Whi5-GFP for the cell shown in (F). Pheromone was added at the 153^rd^ minute, and the cell shown is shmooing at the 390^th^ minute. All Whi5-GFP is expected to be nuclear during pheromone arrest, supporting our use of the corrected (red) trace over the uncorrrected (green) trace, which drops significantly.

The purpose of segmentation and tracking is to extract information about some particular property through time (cf step 21 in the algorithm section). Whereas the segmented phase image allows for the extraction of morphological features such as cell area, minor/major axis, circumference and center point (Figure 6a–c), fluorescent markers can be used to determine dynamics of proteins of interest. To demonstrate the applicability of our algorithm, we investigated cells expressing a C-terminal GFP fusion of the transcriptional inhibitor Whi5 from the endogenous locus. Whi5 is exported from the nucleus during the cell cycle phase G1 (prior to DNA replication) and imported again at the end of the cell cycle, making it an excellent reporter of cell cycle dynamics (Figure 6d,e) [1], [30], [31]. Cells were grown, tracked, and segmented, and a 2D Gaussian function was fit around the peak-GFP signal to determine the dynamics of Whi5-GFP. For a practical example of how this method can be used to characterize cell cycle transitions, see [1].

### Out-of-focus Correction

Even with the use of a hardware-based automatic focus tool such as Definite Focus (Zeiss, Germany), the cell nucleus may still be out of the plane of focus due its movement (Figure 6f). To correct for variation in a nuclear (*e.g.,* Whi5-GFP) signal that are caused by movement of the nucleus relative to the plane of focus, it is possible to use a more constant-brightness nuclear marker as a standard for comparison. Here, we used a histone (Htb2) fused with a red fluorescent protein (mCherry) expressed from the endogenous locus to identify time periods with a consistent decrease in the mean nuclear intensity. Since the mean nuclear intensity, as measured by the Htb2-mCherry signal per nuclear area, is supposed to only increase during S-phase, we assume that, after smoothing, any decrease in the mean nuclear intensity, especially if accompanied by a decrease in Whi5-GFP, indicates an out-of-focus nucleus in G1. In these sections, we correct the Whi5 signal by increasing it in direct proportion to the fractional decrease in the mean nuclear intensity of the Htb2-mCherry signal in the red channel (Figure 6g).

To verify this approach in general, and the use of directly proportional correction in particular, we applied this algorithm to analyze pheromone-arrested cells. Arrested cells are ideal for testing because we expect them to maintain a high level of nuclear Whi5 [reference doncic ‘11] so that variation in the Whi5 signal likely due to nuclear movement. Indeed, when we optimized the coefficient of proportionality to minimize variation in Whi5 levels for individual cells, we have found that the direct proportionality (a coefficient of 1) was close to, and not statistically different from, optimal. We therefore conclude that direct proportionality can be used to correct nuclear signals from distortion due to nuclear movement relative to the plane of focus.

### Conclusion

Recent advances in the automation of time-lapse microscopy and microfluidics greatly facilitate the generation of high quality single-cell data. However, analysis of such data relies on accurate cell segmentation and tracking over many frames. We here present an accurate and easy-to-implement algorithm for automatically segmenting budding yeast phase images over long time-courses. The main principle underlying our algorithm is the application and addition of all possible segmentation thresholds, which results in a very accurate and robust segmentation.

The fundamental challenge when segmenting any object is to segregate between the object of interest and the background. Whereas most segmentation algorithms find an optimal threshold given some segregation method (*e.g.,* filtering, edge detection, watershedding) we here use *all* thresholds using one algorithm: watershedding. This makes the segmentation very accurate, because we capture much more information in the ensemble than could be captured in any one segmentation event. Moreover, although the majority of cells can be segmented with a single optimized threshold, the optimized threshold is rarely able to segment all of the cells, all of the time. Thus, ‘optimal threshold’ methods will necessarily be more error prone as more information is lost when the single threshold is applied.

Our algorithm has several further benefits: It does not require that cells take any particular geometric shape and can therefore segment hyperpolarized and shmooing cells as well as round and oblong ones. The algorithm allows for manual selection of cells in the last time frame, so no time is spent on out of focus cells, buds, dead cells or other uninteresting objects. Our algorithm can segment arbitrarily large colonies, as it segments and tracks cells one-by-one backwards through time.

As a proof of concept, we have also shown how our algorithm can be used as a basis for extracting quantitative dynamic traces of a fluorescent protein translocated from the nucleus in a cell cycle dependent manner. Accuracy in increased when this approach is combined with a subroutine that corrects for nuclear drift. High quality quantification of fluorescent proteins in single cells can later be used to elucidate more complex cellular behavior such as transcriptional dynamics, size control and cell fate decisions [1], [5], [32]. Notably, our algorithm is modular, so that segmentation and tracking (phase) is done by separate subroutines from fluorescence measurement. It is therefore easy to add custom subroutines to detect and quantify specific fluorescent proteins of interest.

The algorithm described here is not limited to yeast phase images because preliminary tests suggest that it can also be applied to bright-field yeast time series and slow moving mammalian cells with distinct boundaries (AD, unpublished data). However, such applications will require extensive modification of the implementation described here, and are beyond the scope of this paper.

Many cellular processes are governed by all-or-none effects, memory, and oscillations. Because these phenomena are masked in bulk assays, their study requires high quality single-cell data. As time-lapse microscopy can be performed by off-the-shelf instrumentation, the bottleneck in the generation of high quality single cell data has become image processing. Here, we applied the simple principle of democratically summing up segmentations for all possible thresholds to develop an efficient algorithm for segmenting and tracking yeast phase images. The simplicity of the underlying idea suggests that this approach could be used to improve single-cell studies in a variety of contexts.

## Materials and Methods

### Algorithm Outline

To facilitate the implementation of our algorithm for segmenting and tracking cells, we here present a point-by-point description accompanied by a graphical representation (algorithm flowchart; Figure 2, also note that variable names are written in *italics*). To users not familiar with programming we have also provided a user friendly GUI that implements our algorithm (see Supporting Information S1 for more details).

1. Set *current cell number* to 1.
2. Set *current time-point* to *first time point* (t_max_).
3. For the *current cell* and *current time*-point, check if the cell still exists against a list of all cells (all cells always exist in the *first time* point).
4. Set the *current segmentation threshold* to 0 and let the *current phase image* be a fixed sub-region of the phase image for the *current time-point* around the location of the cell for the previous time-point (the seed) (Figure 3a). Create an empty image of the same dimensions to be the *next segmentation image*.
5. Increase the *current segmentation threshold* by 1. Note that this threshold is related to the intensity of the pixels in the phase image we are about to segment. We use uint8 images where each pixel can take 2^8^ values ranging from 0 (black) to 255 (white).
6. Use the *current segmentation threshold* to divide the *current phase image* into a binary image of putative ‘cell’ and ‘no-cell’ areas. Pixels in the current phase image below the *current segmentation threshold* are considered to potentially belong to the ‘cell’ category (Figure 3b).
7. Segment the binary image from step 6 using the watershed algorithm. We use the Ferdinand Meyer algorithm via the MATLAB watershed function [33]. Briefly, the watershed algorithm separates the continuous cell or no-cell regions from step 6 into distinct sub-regions (Figure 3c). In practice, this is accomplished by calculating the distance transform of the complement of the binary image defined in step 6 and assigning any pixels that do not belong to any object as minus infinity. Thus a ‘topographic map’ is created for the object to which the watershed algorithm can be applied.
8. To determine whether any such sub-region is part of the cell, score each sub-region as 1 (cell) if three criteria are satisfied: (i) the area overlaps more than 40% with the seed image; (ii) less than 33% of the area consists of non-cell regions as classified in step 6; and (iii) no part of the region is located at the image border. Otherwise, score the sub-region as 0 (no cell). Repeat this for all sub-regions for this cell for the *current segmentation threshold* (Figure 3d).
9. Add the image from step 8, consisting of the scored watershed regions, to the *next segmentation image*.
10. Increase the *current segmentation threshold* by 1 and repeat steps 6 through 9 until the *current segmentation threshold* reaches 256. The *next segmentation image* will thus be a composite image whose pixels are all between 0 and 256 depending on how many watershed segmentations scored it as ‘cell’ (Figure 3e).
11. To penalize cell movements perform a Euclidean distance transformation on the seed image to calculate the distance from each ‘non-cell’ pixel to a ‘cell’ pixel. Next, subtract this image from the *next segmentation image* (Figure 4a,b).
12. To penalize regions with high phase intensity, such as cell boundaries (white regions in Figure 1a), rescale the *current phase image* and subtract it from the *next segmentation image*. Let the resulting image be the *next segmentation image* (Figure 4c).
13. After the transformations in steps 11 and 12, the segmentation scores are normalized by calculating the 90^th^ percentile of the distribution of values for the *next segmentation image*. This value is called the *normalized segmentation score*.
14. Identify pixels from the *next segmentation image* that are above a certain fraction of the *normalized segmentation score* and are present in the seed (this fraction is typically set between 0.2 to 0.4 as we have found these values to work well). This allows for easier acceptance of regions that were previously scored as ‘cell’ in the preceding segmented time point (Figure 4d; upper panels).
15. Identify pixels from the *next segmentation image* that are above a specified fraction of the *normalized segmentation score* (this fraction is normally set 0.2 higher than the threshold in step 14). This allows for the incorporation of previously ‘no-cell’ areas if their score is high enough (Figure 4d; lower panels).
16. Add the results from step 14 and 15 to get a binary image of the *putative segmentation* (Figure 4e).
17. Compare the area for the *putative segmentation* with the seed area. If the area has grown by more than a specified fraction (typically set between 5% and 10%), increase the thresholds used in steps 14 and 15 by 0.2 and the repeat these two steps. Note that this threshold increase is to prevent uncontrolled growth and is only applied once.
18. Confirm that the *putative segmentation* consists of one continuous object, which is larger than the *minimal allowed cell area* (typically 10 pixels). If this condition is not fulfilled, retain the largest sub-object as the new *putative segmentation* and discard all other objects.
19. Next, terminate the segmentation for the current cell if at least one of the following conditions are satisfied: (i) the cell is smaller than the minimal area; (ii) the cell is larger than the maximally allowed area (normally 133% of the area of the largest cell in the initial field of view); or (iii) the increase of size for the last time-point was more than 50% of its previous size. When a cell segmentation is terminated it is marked as non-existing (cf step 3) and its time of disappearance is recorded (this normally corresponds to its birth, if condition (i) is met). Next, increase the *cell number* by one if it is less than the *maximum cell number* and return to step 2 to segment the next cell of interest.
20. If the segmentation has not been terminated, then save the *putative segmentation* as the next seed for this cell.
21. To extract desired information from the segmented cell, call a specific routine to quantify fluorescence and/or morphology. The specific routine called will depend on the type of signal one is trying to extract. Above, we presented an example of the quantification of the nuclear concentration of a fluorescent protein (see also Figure S4 and Supporting Information S1).
22. If there are more time-points to segment, *i.e.*, *current time-point*>*last time point* (which is usually 1) decrease *current time point* by 1 and start over at step 4. Alternatively, if the *last time-point* was reached and the *cell number* equals the *total number of cells*, terminate the program. Otherwise increase *cell number* by one, return to step 2 and continue until all cells are segmented.

### Cell Culture and Microscopy

Segmentation requires that the objects to be segmented remain visible throughout the duration of the experiment. In the case of yeast, this requires that growing colonies be restricted to two dimensions. We used a commercially available flowcell from Cellasic to grow the cells (Table 2) at a temperature of 30°C while being exposed to constantly flowing SCD (synthetic complete media with 2% glucose) at a flow rate of 5psi (∼34 KPa).

**Table 2:** List of strains.

Strain name	Genotype	source
AD4-47c	*MAT* ***a,*** * cln3::LEU2, bar1::URA3 trp1::TRP1-MET3pr-CLN2 WHI5-GFP-kanMX, HTB2-mCherry-spHIS5*	This study
JS136-3c	*MAT* ***a*** * bar1::URA3 trp1::TRP1-MET3pr-CLN2 WHI5-GFP-kanMX HTB2-mCherry-spHIS5*	[1]

All strains used are congenic with W303.

Images were taken every three minutes with a Zeiss Axio Observer Z1 microscope using an automated stage and a plan-apo 63X/1.4NA oil immersion objective. Zeiss Definite Focus hardware was used for automatic focusing. The *WHI5-GFP* strain was exposed for 100 ms using a Colibri LED 470 module and the *HTB2-mCherry* strain was exposed using the Colibri 540-80 LED module, both at 25% power.

### GUI

See Supporting Information S1, Figures S1, S2, S3 and tables S1, S2 for instructions regarding the GUI.

## Supporting Information

Movie S1 **See main text for details.**
(AVI)

Movie S2 **See main text for details.**
(AVI)

Movie S3 **See main text for details.**
(AVI)

Movie S4 **See main text for details.**
(AVI)

Supporting Information S1. (PDF)
