# Supplementary material for: An Algorithm to Automate Yeast Segmentation and Tracking
Source: PLoS One. 2013 Mar 8;8(3):e57970. doi: 10.1371/journal.pone.0057970 (PMC3592893; doi:10.1371/journal.pone.0057970)
Supplement: Supporting Information S1. — (PDF) [file pone.0057970.s005.pdf]

An algorithm to automate yeast segmenting and tracking

Graphical User Interface Manual

Installation:

We implemented a graphical user interface (GUI) for our algorithm in MATLAB. The GUI consists of two parts:

1. Seeding the segmentation, called *CTseed*
2. Segmenting and tracking, called *CTtrack*.

To install the program, either copy the files to the default Matlab folder or add their directory to Matlab's search paths. To run successfully, the GUI requires the MATLAB Image Processing and Statistics toolboxes. The GUI should work with any recent Mat version (we have tested it with versions R10 and R11).

Before tracking:

The GUI uses images from time-lapse experiments as input variables. To facilitate segmenting and tracking, all images belonging to the same experimental field of view (position) should be in a single directory. Moreover, images from each channel should have a unique identifier tag. In our example, we use phase images that are tagged with "c1" and GFP images tagged with "c4". Although the GUI likely supports all image formats, and the Matlab '*imread*' function can handle BMP, JPEG, PNG, CUR, JPEG 2000, PPM, GIF, PBM, RAS, HDF4, PCX, TIFF, ICO, PGM and XWD, we have so far only used JPG and TIFF formats.

Note that the GUI detects phase and fluorescently labeled images based on their unique identifiers. Therefore, the tag identifying the channel cannot be used as part of the image name. For instance, if the GFP has an identifier "c4", and if all the names of images contain a string containing "c4" such as "Pac4 ", then the program will fail to properly select the GFP images. Furthermore, all images should be named in chronological order for the GUI to detect them (see Figure S1, 'image list' for an example).

Seeding step:

To track individual cells, an initial segmentation template made from the last frame of interest (*first time point*) is required because the algorithm segments backwards through time. The GUI subroutine *CTseed* accomplishes this by allowing for manual selection and segmentation of the *first time point*. To open this part of the GUI, either type *CTseed* in the Matlab command line or double click on the *CTseed.fig* file after specifying the image locations as described above.

To segment one or several fields of view in a pipeline, click on the '*Browse'* button and select all folders containing the exported images to be segmented and tracked (Figure S1). A list of individual images will appear in the box on the right if they loaded correctly. Next, enter the unique channel identifiers and the last frame to be segmented and click on the '*OK*' button to start the manual selection (Figure S1). A window will appear to manually choose a threshold for the initial segmentation (Figure S2, and Figure

1D in the main text). The left panel shows the seed phase image in which the pixel intensity is displayed using a color scale. The right panel contains the binary version of the left image after the threshold defined by the user was applied. If needed, the threshold can be adjusted using the slider tool. Once a reasonable threshold is selected, press '*Done*'. Next, three windows will appear: one with the putative segmented image, one for selected cells (initially black = empty), and one with the original phase image (Figure 1A,B,D in main text).

To select cells of interest, move the crosshairs that appear in the segmented image (Figure 1B in the main text) to a cell of interest and press 's' ('select'). If the cell is not segmented properly its area can be manually corrected using the left and right mouse buttons to remove and add regions to the cell area, respectively (Figure 1C in the main text). Note that each selected cell has to be completely disconnected from all nearby cells. The size of the add/remove cell area cursor can be changed using '+' and '-'. After selecting the cells of interest in a particular field of view, press 'esc' to save and move on to the next field of view. The seeding algorithm automatically fills and removes interior holes and lines as long as they are not connected to the cell's exterior (Figure 1C,D in the main text).

Tracking step:

After seeding, cells can be segmented and tracked using a GUI routine called *CTtrack*. To open *CTtrack*, double click on the *CTtrack.fig*-file or type *CTtrack* in the Matlab command line. The current directory is displayed above the list box that contains the list of items within the current directory (Figure S3). To browse across the directory hierarchy, double click on '**..**' or '**.**' as shown in Figure S3. To select fields of view to be segmented, select the seed files by clicking on them and then click the '>>>>' button. Similarly, the selected seed file in the right list box can be removed from the list by clicking on the '<<<<' button.

To segment and track the cells, a limited set of parameters has to be specified. For a detailed explanation of these parameters see Table S1 below. Note that most parameters remain constant from experiment to experiment.

Parameter name	Typical value	Explanation
Max size vs largest cell	1.33	How much larger (fraction) pixel area a cell can be compared with the final image. A value of 1.33 is equivalent to 33% larger.

max area increase per tp	0.075	Maximal allowed area increase before switching to higher threshold. Units are in the fraction of previous area. A value of 0.075 represents a 7.5% increase
higher threshold	0.4	Segmentation threshold for regions that were not part of the cell in the previous time point (cf Figure 4D in the main text)
lower threshold	0.2	Segmentation threshold for regions that were part of the cell in the previous time point(cf Figure 4D in the main text)
threshold increase factor	0.2	Change of *higher* and *lower* thresholds in the case of a large change in area
phase subtraction factor	1.2	Factor that determines how much of the phase image we subtract from the segmentation
min cell size	10	Minimal allowed cell size in pixels. Note that this variable will scale with the image resolution used (we used 512x672 resolution imaged in our examples).
cell margin	12	Margin around the cell from the previous segmentation, for current segmentation in pixels

Table S1: Segmentation and tracking parameters. These parameters work well for segmenting growing cells. Note that the exact values for some of the variables might need to be changed depending on the resolution, rate of sampling, or cell morphology.

Next, choose adequate display options to monitor progress during tracking and define a save directory by clicking on the '*save*' button (Figure S3). Finally, click the '*run*' button to start segmenting and tracking.

Description of the output file:

The output will be saved as a MATLAB '.mat' file. The file contains an ***all_obj*** structure class variable, which contains the tracking and segmentation data (see Table S2).

Parameters in ***all_obj***	Explanation
all_obj.cells	Matrix whose dimensions correspond to the dimensions of each image times the total number of time points. *I.e.*, if the image resolution is 'x_size X y_size' and the number of time points are 'no_tp' then all_obj.cells is (x_size X y_size X no_tp). This matrix contains the segmented images for all time points and cells. Images are ordered such that the background equals 0 and the cells are numbered from 1 to the number of cells (see Figure S4).
all_obj.cell_area	Matrix whose dimensions are the number of cells and the number of time points. Its

	value is the cell area in pixels for each cell and time point. Note that this is an example of a morphology based metric calculated from *all_obj.cells* (see Figure 6A in main text)

Table S2: Segmentation output

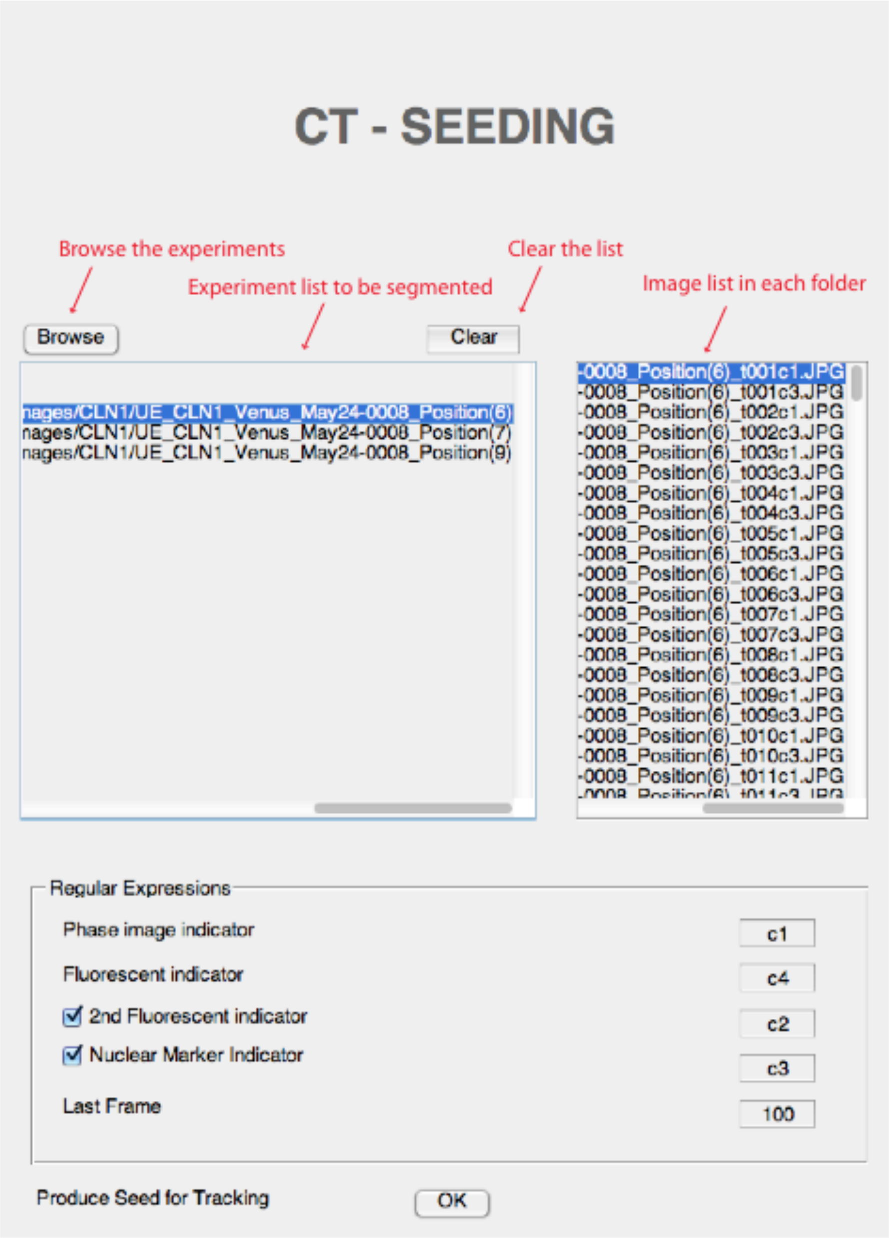

Figure S1: The first part of the GUI, called *CTseed* is designed to seed cells for segmentation and tracking. Example folders loaded (list in left box) and their contents (list in right box) are shown. Channels displayed are 'c1' for phase images and 'c2' for a fluorescent channel, 'c3' is for a nuclear fluorescent marker.

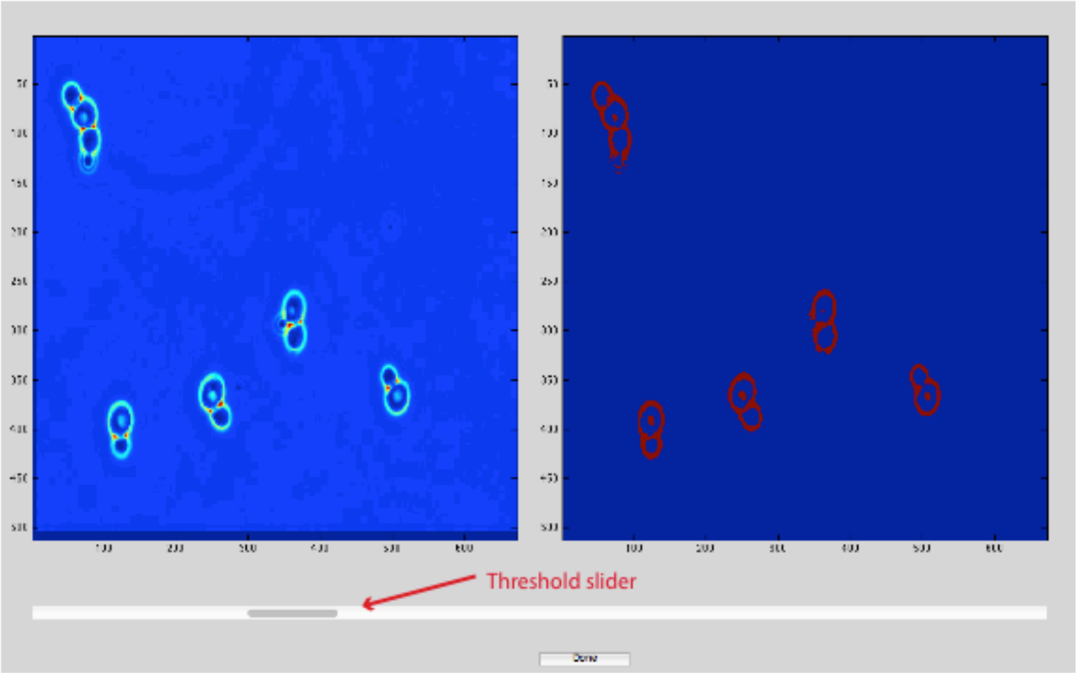

Figure S2: The GUI for selecting the initial seeding threshold. The right panel shows the binary image produced after the threshold is applied. The threshold level can be adjusted by moving the slider.

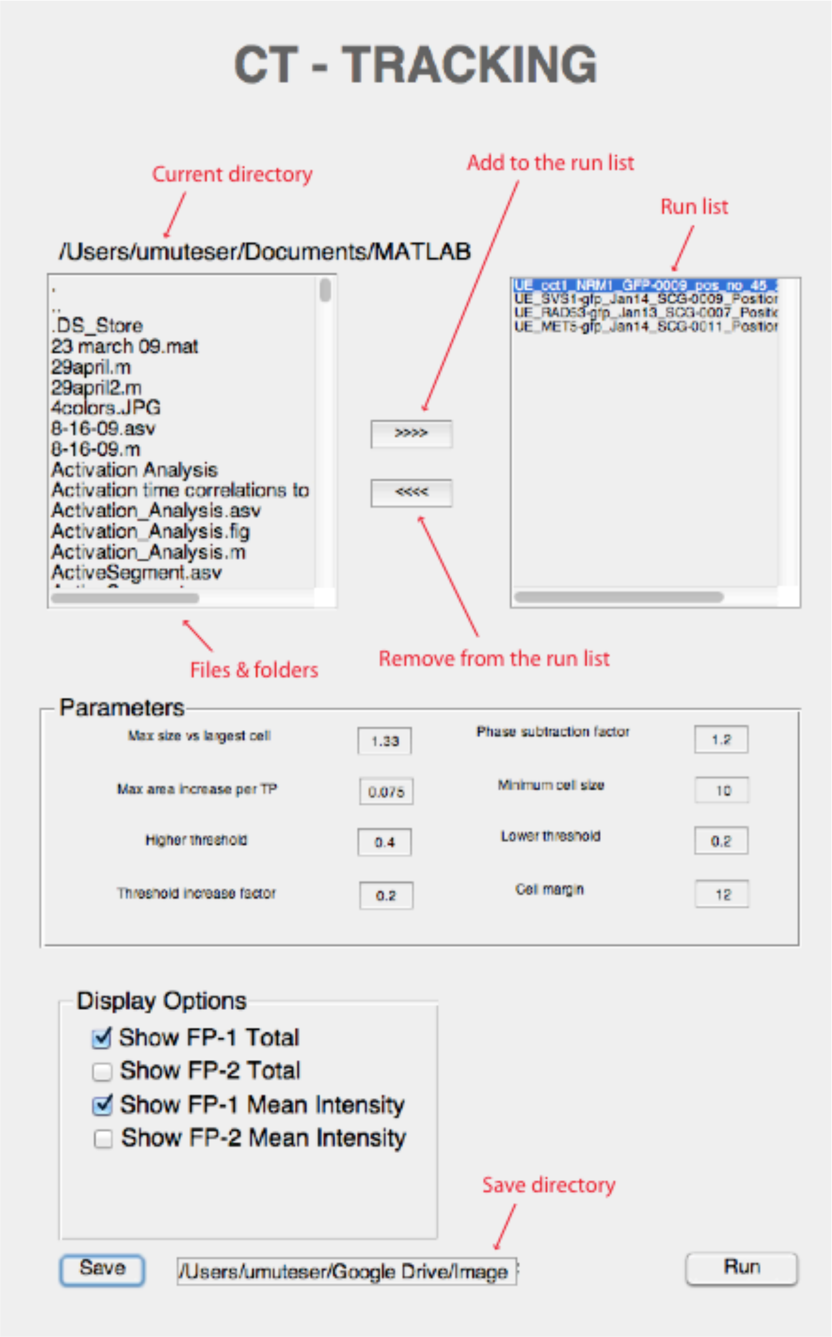

Figure S3: Tracking and segmenting cells is performed by the second GUI subroutine called *CTtrack*, which builds a pipeline of several fields of view and automates the segmentation and tracking. The current directory is shown above the list in the left box. To load seeds produced by the *CTseed* routine click on the right arrows '>>>>>'. Loaded seeds appear in the box on the right hand side. Below are parameters that can be adjusted to optimize segmentation (see text).

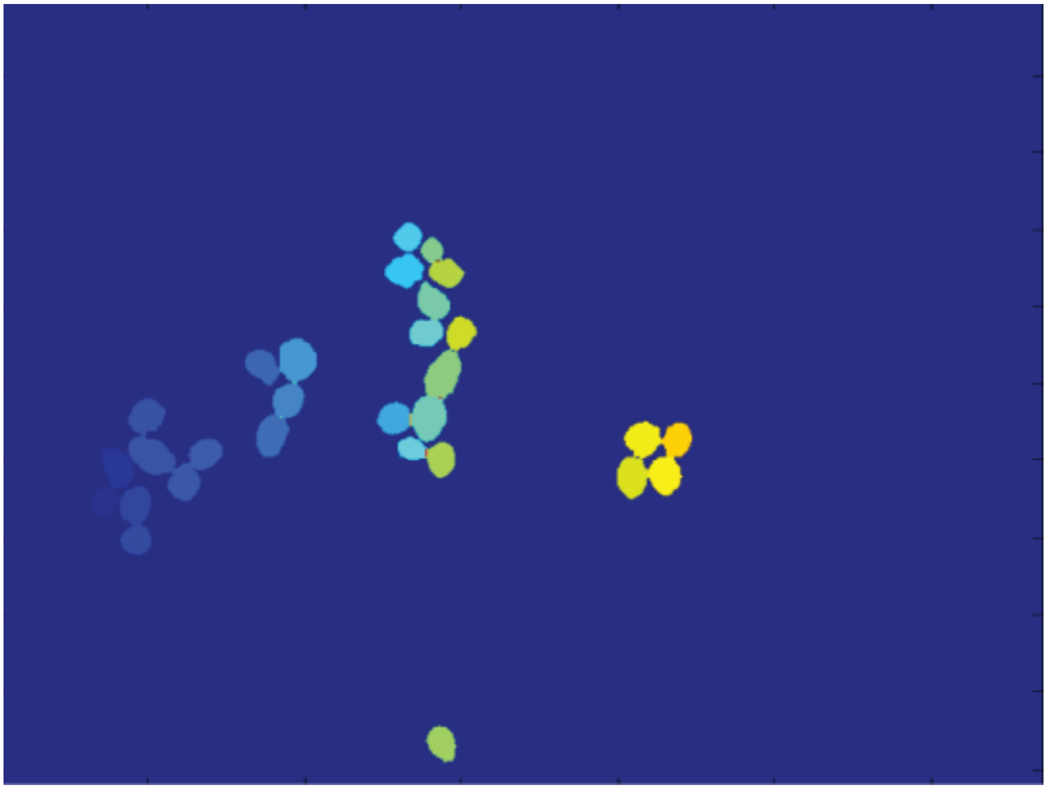

Figure S4: An example view in ‘*all_obj.cells*’ of segmented cells plotted using the ‘*imagesc*’ function in MATLAB (cf Figure 1E I in the main text). Note that all cells are ordered from left to right, which is denoted here using color gradation.
